# The Evaluation of ABO Antibody Titers and Effect of Physical and Lifestyle Factors in Single Donor Platelets From O Blood Group Donors: A Cross-Sectional Study

**DOI:** 10.7759/cureus.95201

**Published:** 2025-10-22

**Authors:** Ramu Thakur, Yogesh Pawde, Bhanupriya Mujhalda, Ashok Yadav, Imlimenba Walling

**Affiliations:** 1 Immunohematology and Blood Transfusion, Mahatma Gandhi Memorial (MGM) Medical College, Indore, IND

**Keywords:** alcohol consumption, anti a and anti b antibodies, blood group o, diet, ig m and ig g, platelet transfusion compatibility, single donor platelets (sdp), smoking, tube method titration

## Abstract

Introduction: Transfusion of single-donor platelets (SDP) from O donors to non-O recipients poses a significant risk of hemolytic reactions due to high-titer anti-A and anti-B antibodies in donor plasma. These antibodies bind to A and/or B antigens on recipient red blood cells, potentially causing an immune response and hemolysis. The primary objective of this cross-sectional study is to quantify IgM and IgG anti-A and anti-B titers in 'O' blood group single donor platelets (SDP). The secondary objective is to assess the influence of donor demographic, physical, and lifestyle factors on antibody levels to guide safe transfusion practices.

Methods: A cross-sectional study was conducted on 100 group O SDP donors at a tertiary hospital from December 2022 to November 2023. Antibody titration was performed using the tube method with serial dilutions. The IgM and IgG titers were assessed by two blinded observers. Donor characteristics such as age, gender, diet, smoking, alcohol use, and BMI were recorded via questionnaire. Associations with antibody titers were analyzed.

Results: More donors had high anti-A IgM titers (≥64) versus anti-B IgM (43% vs. 26%, p = 0.0053). Anti-A IgG ≥128 was more common than anti-B (56% vs. 38%, p=0.002). Anti-A IgM and IgG titers remained stable across age groups, while anti-B IgM showed a weak negative correlation with age (ρ = -0.21, p = 0.04); anti-B IgG was unchanged across ages (ρ = -0.08, p = 0.42). Females had higher antibody titers than males (p<0.05). Vegetarian donors had a higher proportion of anti-A IgM levels≥64 than mixed-diet donors (56.86% vs. 28.57%, χ² = 8.10, p = 0.004). The IgG titers did not differ by diet. Lower IgM and IgG levels were found in smokers versus non-smokers (p<0.05). Alcohol consumption correlated with lower anti-A/B IgG levels (p<0.02) but not with IgM titers. Weak correlations existed between ABO antibody titers and BMI (range: r = -0.08 to 0.08, all p > 0.38). Spearman analysis showed no association between platelet yield and ABO antibody titers (anti-A IgM, anti-A IgG, anti-B IgM, anti-B IgG), with correlations near zero (ρ = -0.06, -0.05, -0.08, -0.02) and p > 0.05.

Conclusion: Group O SDP donors show higher anti-A than anti-B antibody levels, with titers influenced by gender, diet, and lifestyle factors. Routine antibody titer assessment may help identify safer platelet units for non-identical ABO transfusions, optimizing safety and utilization in emergencies.

## Introduction

The ABO blood group system is crucial for transfusion compatibility. Type O donors possess ABO antibodies that interact with antigens on RBCs from other groups, potentially causing hemolytic reactions. Single-donor platelets (SDP) from type O donors may have high ABO antibody levels. Platelet transfusions should use ABO-identical platelets [1].

Single donor platelets contain high plasma volume, increasing intravascular hemolysis risk. Consequences range from mild to severe, depending on donor antibody titers and recipient characteristics [2]. Measures must prevent intravascular hemolysis through careful donor-recipient selection to avoid ABO incompatibility. The ABO-compatible platelets should be used when feasible. However, this is often impossible due to scarce donor platelets. In such cases, platelets with low ABO antibody levels are crucial [3]. This is challenged by the frequent unavailability of ABO-identical platelets and balancing inventory with safer transfusion needs. Due to limited group-specific platelets, O blood type SDP can be given after antibody titer and compatibility testing [4]. While studies have quantified ABO antibodies in O group SDP, there is limited understanding of how physical and lifestyle factors such as diet, smoking, alcohol, and body mass index (BMI) influence these titers [5]. Natural anti-A and anti-B antibodies arise from exposure to microbial and dietary carbohydrate antigens mimicking ABO glycans; gut microbes with A-/B-like glycans stimulate B-cells producing these antibodies [6] [7].

The BMI affects ABO antibody titers through multiple pathways. Inflammation caused by obesity impairs B-cell function and reduces antibody production. Elevated adiposity disturbs immune cell metabolism and reduces humoral responses. Changes in gut microbiota may alter antigen exposure. Studies of ~7,450 Japanese blood donors found that higher BMI correlated with lower anti-A and anti-B titers (in blood group O donors) [8,9]. Diet influences gut microbiome, altering bacteria and carbohydrates that affect mucosal and systemic antibody responses [6,10]. Studies show higher odds of elevated anti-A/anti-B titers in donors with vegetarian diets [5,11]. Smoking impairs antibody titers by reducing antigen-presenting cell function and T-cell signalling for B-cell activation [12]. Alcohol affects immunoglobulin levels: heavy drinking elevates IgA, while moderate drinking may reduce IgG and IgM through gut antigens and inflammation [13]. High platelet yields contain larger plasma volumes with ABO antibodies, increasing hemolysis risk. Mitigation includes donor screening, platelet additive solutions (PAS), or ABO-identical use. Although PAS decreases the amount of antibodies, titer screening continues to be more cost-effective. Research is required to determine if the platelet yield from donors can predict the antibody load [3,14].

This study aimed to quantify IgM and IgG anti-A and anti-B antibody titers in the O blood group SDP using the tube method and assess the impact of physical and lifestyle factors. Understanding antibody levels helps clinicians make informed decisions about ABO-mismatched platelet use. This study hypothesized that physical and lifestyle factors influence ABO antibody titers in O group SDP, affecting transfusion practices.

## Materials and methods

Study design and setting

A cross-sectional study was carried out at the blood center of a tertiary care hospital in Indore, Madhya Pradesh, India, between December 2022 and November 2023 (12-month duration). The study was approved by the Institutional Ethics Committee of Mahatma Gandhi Memorial (MGM) Medical College and Maharaja Yeshwantrao (MY) Hospital, Indore (approval no. EC/MGM/DEC-22/18). All donors who participated in this study provided written informed consent.

Study population

A total of 100 voluntary O group single-donor platelet (SDP) donors were included in the study using simple random sampling, and written informed consent was obtained before donation. The sample size was calculated using the following formula where p is the prevalence of blood group O (31.14%) in Madhya Pradesh of India [15], q=100−p, and d=10% margin of error:

$n = \frac{4 \times p \times q}{d^{2}}$

$n = \frac{4 \times 31.14 \times 68.06}{10^{2}} = 85.77 \approx 100$

**Figure 1:**
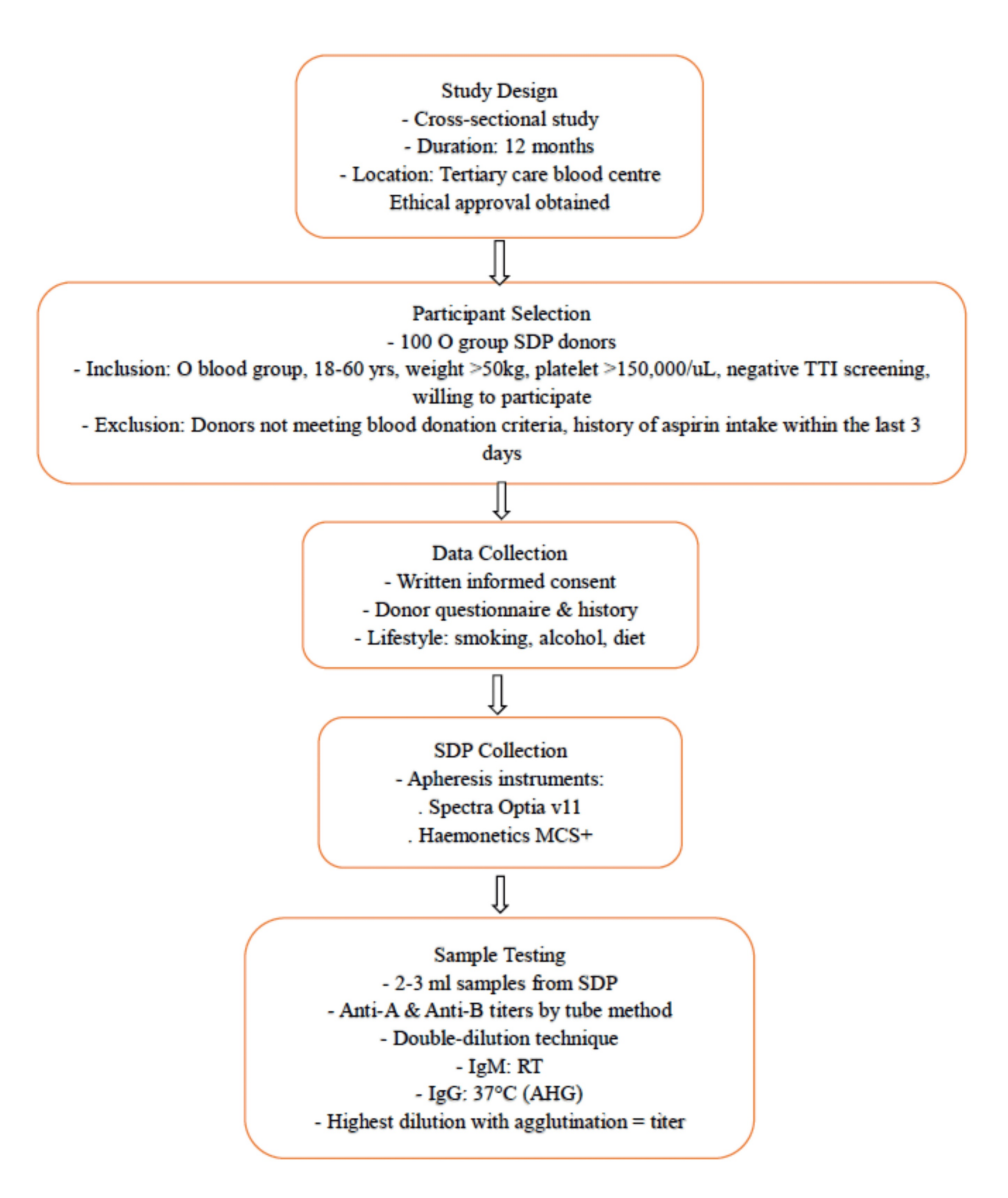
Study design and testing protocol TTI: Transfusion transmitted infection, AHG: Anti-human globulin, SDP: Single donor platelets, RT: Room temperature Spectra Optia v11 (Terumo Blood and Cell Technologies, Lakewood, CO, USA); Haemonetics MCS+ (Haemonetics Corp., Boston, MA, USA)

The study protocol is detailed in Figure 1. The selection and deferral of blood donors were performed per the guidelines established by the National Blood Transfusion Council (NBTC) and the Directorate General of Health Services (DGHS), India [16]. The inclusion criteria were age between 18 and 60 years, weight of at least 50 kg, hematocrit of ≥38%, pre-donation platelet count of at least 150 × 10^9/L, and negative screening for transfusion-transmissible infections (TTI) for HIV, hepatitis B surface antigen (HBsAg), hepatitis C virus (HCV), malaria parasites, and syphilis. Participants provided informed consent via a blood donor consent and questionnaire form (see Appendix A). Data on demographic details, physical and lifestyle factors, and dietary habits were collected through a separate questionnaire (see Appendix B).

SDP procedure and sample collection

Platelet collection was conducted using the Spectra Optia version 11 (Terumo Blood and Cell Technologies, Lakewood, CO, USA) and Haemonetics MCS+ (Haemonetics Corp., Boston, MA, USA) apheresis systems that were employed interchangeably depending on the availability of machines and sterile disposable kits. Donor demographic and hematological parameters (age, sex, weight, height, hematocrit, platelet count, and desired platelet yield) were entered into the system, which then automatically calculated the estimated blood volume, process volume, target cycles, and predicted procedure duration. These calculated values were verified and, if required, adjusted manually by the operator to ensure donor safety and optimal collection parameters.

Venipuncture was performed under aseptic precautions after skin disinfection, and initial diverted blood was collected in a diversion pouch. The anticoagulant citrate dextrose solution A (ACD-A) was initially set to the machine's default inlet: ACD ratio but was adjusted to a range of 1:10 to 1:15 during the procedure to ensure donor safety and optimal platelet collection. Throughout the procedure, donors were monitored for any adverse reactions.

On completion, the product bag was labeled with the unit number, collection date, expiry date, and blood group, and barcoding was applied for traceability. A 2 ml to 3 ml sample of SDP was obtained through the attached sample pouch, sealed using a sterile tube sealer, and transferred into a sterile test tube for antibody titration.

Antibody titration

Anti-A and anti-B antibody titers (IgM and IgG) were measured using the conventional tube agglutination method following DGHS guidelines [16]. Each titration was performed in duplicate, and readings were independently assessed by two observers. Observers were blinded to donor characteristics to minimize bias. Samples collected in test tubes were used for two independent sets of serial two-fold dilutions in normal saline (starting from 1:1, 1:2, 1:4, and 1:8 and ranging up to 1:1024), with further dilutions performed if agglutination persisted at the highest dilution to accurately determine the endpoint titer. For testing, 3% to 5% suspensions of freshly prepared, well-typed group A1 and group B red cells were used.

For IgM titration, equal volumes of serum dilution and red cell suspension were mixed in labeled tubes, centrifuged at 1000 rpm for one minute, and agglutination was read macroscopically. The IgG titration was performed in parallel by incubating dilutions at 37°C for 30 to 45 minutes, followed by three saline washes to remove unbound antibody. After adding polyspecific antihuman globulin (AHG) reagent, the tubes were centrifuged, and agglutination was recorded. Agglutination reactions were assessed by trained personnel using uniform grading criteria (DGHS) to maintain consistency across observations [16]. Coombs control cells were added to all negative AHG tubes to confirm validity. The endpoint titer was defined as the highest dilution showing ≥1+ macroscopic agglutination, and results were expressed as the reciprocal of this dilution (e.g., 64). Additional dilutions were performed if agglutination persisted at the highest prepared dilution.

Statistical analysis

Data were entered and organized in Microsoft Excel (Microsoft Corp., Redmond, WA, USA). Data distribution was checked for normality, and parametric or non-parametric tests were applied as appropriate. Data analysis was conducted using SPSS Statistics version 29.0.2 (IBM Corp., Armonk, NY, USA). The two-sided t-test with a significance level of 0.05 was employed to assess the average antibody titer levels, after testing for equality of variances between groups to ensure appropriate application of the test. Spearman's correlation coefficient (ρ) was used to assess the relationship between antibody titers and various variables such as age, BMI, and platelet yield. The relationship between antibody titers and various factors was evaluated using the chi-square test (χ²), including smoking, gender, alcohol consumption, and dietary habits. A p-value < 0.05 was considered significant.

## Results

In this study, out of 100 SDP donors, 90% were male and the remaining 10% were female. The age range of the donors was between 18 and 52 years, with the median age of the participants being 31 years (IQR, 26-33 years). The average height and weight were 169.4 cm and 73.8 kg, respectively. The BMI was 25.7 kg/m². The average platelet count was 285.6 × 103/µl, and the mean platelet yield was 3.9×10¹¹.

The definitions of 'high-titer' platelet units vary considerably between centers; nevertheless, many classify IgM anti-A and anti-B as high at titers ≥1:64 [5], whereas IgG anti-A and anti-B are considered high at titers ≥1:128 [17]. Titers among donors ranged from 4 to 1024 (Table 1). For IgM antibodies, most donors had titers of 16 to 64, with 74% for anti-A and 71% for anti-B. Smaller groups showed titers of 4 to 8 (18% and 21%), while some had titers from 128 to 256 (8% each). No donors had IgM titers of 512 or higher. Anti-A IgG titers were mainly in the 128 to 256 range (51%), followed by 16 to 64 (43%), with 5% reaching 512 to 1024 and 1% at 4 to 8. Anti-B IgG titers occurred mostly at 16 to 64 (61%) and 128 to 256 (34%), with 4% at 512 to 1024 and 1% at 4 to 8.

**Table 1:** Frequency of IgM and IgG Type of anti-A And anti-B Titer among O blood group SDP donors SDP: Single donor platelets

Titer range	Anti-A IgM	Anti-B IgM	Anti-A IgG	Anti-B IgG
4-8	18	21	1	1
16-64	74	71	43	61
128-256	8	8	51	34
512-1024	0	0	5	4
Total	100			

As shown in Table 2, 43% of the donors had anti-A IgM titers of 64 or higher, compared to 26% for anti-B, while 57% had anti-A IgM titers below 64, and 74% had anti-B IgM titers below this level (p = 0.0053). A significantly larger proportion of donors (56%) had anti-A IgG titers ≥ 128, in contrast to 38% for anti-B (p = 0.002). On the other hand, 62% of donors had anti-B IgG titers less than 128, compared to 44% for anti-A.

**Table 2:** Prevalence of antibody titer among O blood group SDP donors SDP: Single donor platelets

Antibody titer	Anti-A IgM (n=100)	Anti-B IgM (n=100)	p value
Titer ≥64	43 %	26%	0.0053
Titer <64	57%	74%	
	Anti-A IgG (n=100)	Anti-B IgG (n=100)	p value
Titer ≥128	56%	38%	0.002
Titer <128	44%	62%	

Correlation of gender with ABO antibody titer

For IgM antibodies, 38.8% of males had anti-A titers ≥64 compared to 80% of females (χ² = 6.20, p = 0.01), while anti-B IgM titers ≥64 were in 23.3% of males and 50% of females (χ² = 3.32, p = 0.06). For IgG antibodies, 51.1% of males had anti-A titers ≥128 versus 90% of females (χ² = 5.49, p = 0.01), and anti-B IgG titers ≥128 were in 31.1% of males and 100% of females (χ² = 18.12, p = 0.00002). The results indicate elevated anti-A and anti-B antibody titers in females, but the gender imbalance may affect findings. Further research with a more gender-balanced cohort is necessary to substantiate these findings.

Correlation of age with ABO antibody titer 

The median titers of anti-A and anti-B antibodies (IgM and IgG) across age groups are shown in Table 3. Anti-A IgM and IgG titers remained constant across ages (32 and 128, respectively), with no correlation with age (ρ = -0.15, p = 0.13 for IgM; ρ = -0.08, p = 0.41 for IgG). Anti-B IgG titers were stable across age groups (median 64, ρ = -0.11, p = 0.27). Anti-B IgM showed a decline with age, highest in the 18 to 25 years age group (32) and lower in other groups (16), showing a weak negative correlation with age (ρ = -0.21, p = 0.04). While anti-A antibody levels remain unaffected by age, anti-B IgM may decrease in older donors. The IgM titers decrease with age due to B-cell function decline, while IgG titers remain stable through immune memory [8].

**Table 3:** Correlation of antibody titer with age

Antibody	Median titer by age group (years)				Spearman’s correlation (ρ)	p-value
	18 to 25 (n=21)	26 to 33 (n=51)	34 to 40 (n=21)	>40 (n=7)		
Anti-A IgM	32	32	32	32	-0.15	0.13
Anti-A IgG	128	128	128	128	-0.08	0.41
Anti-B IgM	32	16	16	16	-0.21	0.04
Anti-B IgG	64	64	64	64	-0.11	0.27

Association of ABO antibody titer with dietary habits

**Figure 2:**
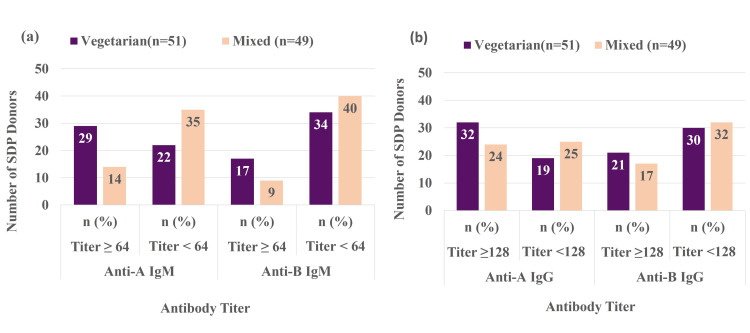
Association of antibody titer with diet (a) Association with IgM titer; (b) Association with IgG titer SDP: Single donor platelets

Figure 2 panel (a) shows antibody titers by dietary habits. In the vegetarian group, 29 (56.86%) donors had anti-A IgM titers ≥64, while 22 (43.13%) had titers <64. In the mixed-diet group, 14 (28.57%) had titers ≥64, and 35 (71.42%) had titers <64. The results (χ²=8.1, p=0.004) were significant, showing variability between groups. For anti-B IgM, 17 (33.33%) vegetarian donors had titers ≥64, while 34 (66.66%) had titers <64. Plant-based diet carbohydrates and gut microbes may enhance anti-A and anti-B antibody production [6,7], while microbiome variations indicate environmental factors affect antibody titer heterogeneity [10].

In the mixed-diet group, nine (18.36%) participants had a titer ≥64, while 40 (81.63%) had a titer <64 (χ² = 2.90, p = 0.08). Figure 2 panel (b) shows that 62.75% of vegetarian donors had anti-A IgG titers ≥128, while 37.25% had titers <128. 41.17% had anti-B IgG titers ≥128, and 58.8% had titers <128 (χ²=1.92, p=0.16), indicating no correlation between diet and IgG antibody levels. Among mixed-diet donors, 48.97% had anti-A IgG titers ≥128 and 51.02% had titers <128, while 34.69% had anti-B IgG titers ≥128 and 65.3% had titers <128 (χ²=0.44, p=0.50).

Our findings suggest dietary habits influence ABO antibody strength, as vegetarians showed higher anti-A IgM titers than mixed-diet donors. This aligns with evidence that dietary antigens and gut microbiome affect isoagglutinin formation. Studies show links between ABO blood group, microbiota, and immune responses [6,7,10].

Effects of smoking and alcohol on ABO antibody titer

**Figure 3:**
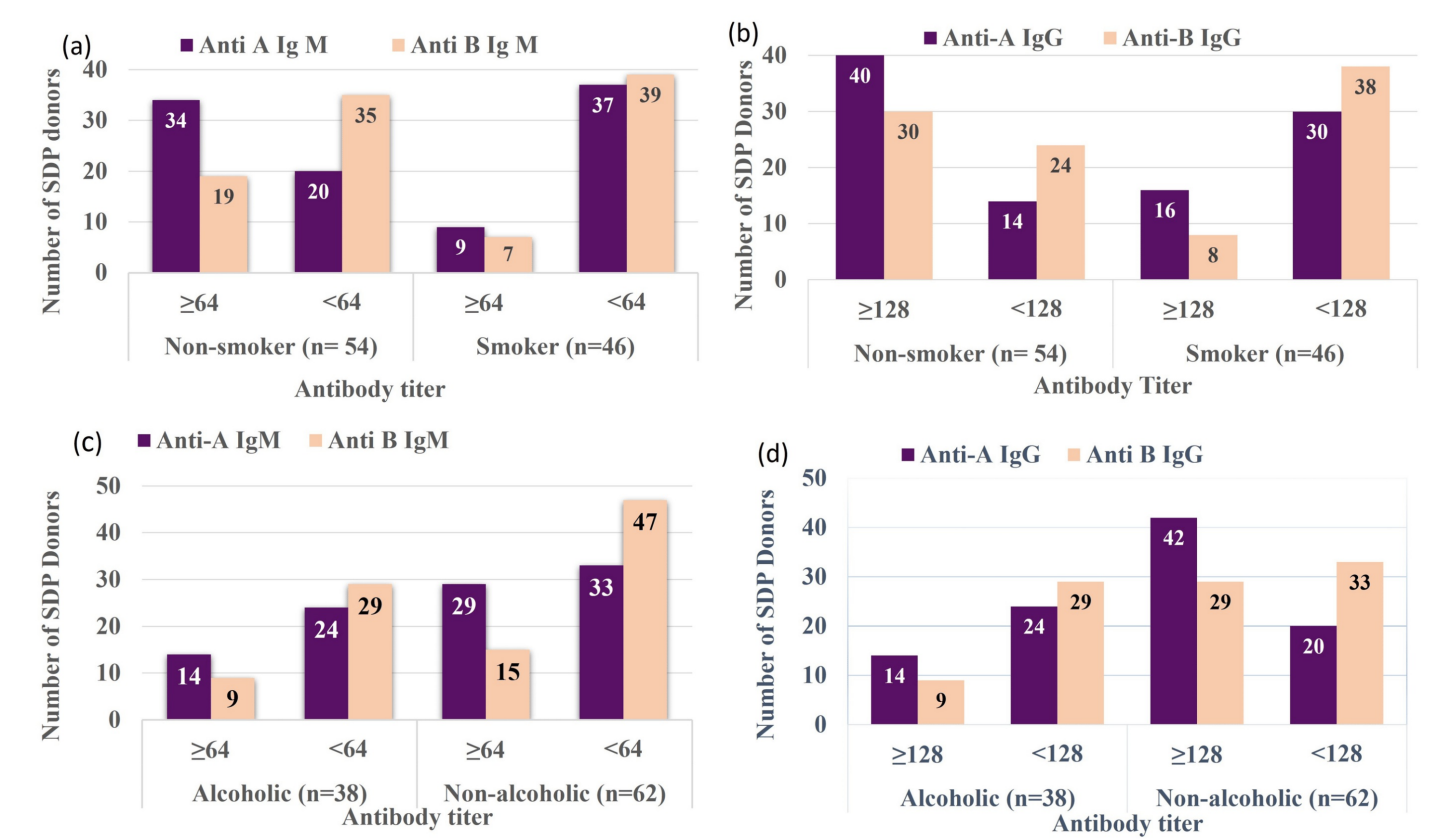
Association of antibody titer with smoking and alcohol (a) Anti-A and anti-B IgM titer associated with smoking; (b) Anti-A and anti-B IgG associated with smoking; (c) Anti-A and anti-B IgM associated with alcohol; (d) Anti-A and anti-B IgG associated with alcohol SDP: Single donor platelets

Among non-smokers (Figure 3 panel (a)), a higher proportion had elevated titers (≥64) of anti-A IgM (62.96%) and anti-B IgM (35.18%) compared to smokers, who showed lower titers (<64) (80.43% and 84.78%). The chi-square test showed a significant association between smoking status and both anti-A IgM (χ²=19.08, p=0.00001) and anti-B IgM (χ²=5.14, p=0.02), indicating non-smokers have higher IgM antibody titers than smokers.

Figure 3 panel (b) shows non-smokers had a higher proportion of anti-A IgG titers ≥128 (74.07%) than smokers (34.78%) (χ²=15.5, p=0.00007). For anti-B IgG, more non-smokers (55.55%) had titers ≥128 than smokers (17.39%), with smokers showing a higher percentage (82.61%) of titers <128 (χ²=15.3; p=0.00008).

In the alcoholic group, 36.84% showed anti-A IgM titers ≥64, compared to 46.77% in the non-alcoholic group (χ²=0.94, p=0.33). Anti-B IgM levels were not statistically significant (χ²=0.003, p=0.95) (Figure 3 panel (c)). For anti-A IgG, fewer alcoholics had titers of ≥128 (36.84%) than non-alcoholics (67.74%), as shown in Figure 3 panel (d) (χ²=9.1, p=0.002). Anti-B IgG titers differed significantly (χ²=5.3, p=0.020) between alcoholics (23.68% ≥128) and non-alcoholics (46.77% ≥128).

Correlation of BMI and ABO antibody titer

The median titers of anti-A IgM (32), anti-A IgG (128), anti-B IgM (16), and anti-B IgG (64) were identical across normal, overweight, and obese donors (Table 4). Spearman's analysis showed no correlation of BMI with anti-A IgM (ρ = -0.097, p = 0.337, 95% CI (-0.28, 0.09)), anti-A IgG (ρ = -0.073, p = 0.469, 95% CI (-0.26, 0.12)), or anti-B IgG (ρ = -0.092, p = 0.362, 95% CI (-0.27, 0.10)). The BMI showed a weak negative correlation with anti-B IgM titers (ρ = -0.205, p = 0.041, 95% CI (-0.38, -0.01)). Higher BMI may impair B-cell function, reducing IgM antibody production. These antibodies are crucial for the immune response. The BMI correlation with anti-B IgM suggests reduced antibody production with increased body fat, while IgG responses remain unchanged [8,9].

**Table 4:** Median ABO antibody titer and correlation across BMI groups

Antibody	Median titer by BMI group			Spearman's correlation (ρ)	p-value	95% CI
	Normal	Overweight	Obese			
Anti-A IgM	32	32	32	-0.097	0.337	-0.28, 0.09
Anti-A IgG	128	128	128	-0.073	0.469	-0.26, 0.12
Anti-B IgM	16	16	16	-0.205	0.041	-0.38, -0.01
Anti-B IgG	64	64	64	-0.092	0.362	-0.27, 0.10

The Spearman correlation analysis showed no statistically significant association between platelet yield and any of the four ABO antibody titers (anti-A IgM, anti-A IgG, anti-B IgM, anti-B IgG). The correlation coefficients were minimal (ρ = -0.06, -0.05, -0.08, -0.02), indicating an absence of meaningful correlation. The corresponding p-values (0.57, 0.65, 0.45, 0.83) were not significant.

## Discussion

In O group SDP donors, the level of anti-A antibodies was higher than that of anti-B for both IgM and IgG, as O group donors generate stronger immune responses against A antigens [1,18]. This heightened response involves both innate and adaptive immunity. The body deletes cells that would produce antibodies against its own blood group, limiting responses to similar antigens on pathogens [19]. In group O individuals, the absence of A and B antigens allows the immune system to produce antibodies against both, particularly anti-A. Innate immune lectins such as galectins -4 and -8 further enhance defense by recognizing blood group antigens on pathogens and supporting adaptive responses. These mechanisms help explain the stronger anti-A response in group O donors, which may increase the risk of hemolytic reactions when their platelets are transfused to A or AB recipients. European policies for preventing hemolytic transfusion reactions following ABO non-identical platelet transfusions have established critical titers of >1:64 for IgM, >1:256 for IgG, and >1:16 for hemolysins. These cut-offs, though not based on strong clinical outcome data, are widely used across many countries [20,21]. However, there are no Indian guidelines specifying critical titers.

This research examined donor-related factors influencing ABO antibody titers. Age had minimal impact, with anti-A titers consistent across age groups, though anti-B IgM levels slightly decreased with age. Dietary habits affected antibody production, with vegetarian donors showing higher anti-A IgM titers than those with mixed diets. Lifestyle factors showed significant associations: non-smokers had higher IgM and IgG titers than smokers, and alcohol consumption was linked to reduced IgG levels. The BMI showed a weak negative correlation with anti-B IgM, while platelet yield showed no correlation with antibody titers. 

Romphruk et al. documented titers from 4 to 1024, noting anti-B predominance in Thai donors, while our Indian donors showed stronger anti-A responses, with many having titers of ≥64 [22]. Bazigau et al. reported median anti-A titers of 64 and anti-B titers of 32 [23]. Tendulkar et al. found most O group donors had titers of ≥64 for both antibodies, with a significant difference between anti-A and anti-B (p < 0.001) [18]. These results suggest anti-A predominance in O group SDP donors is influenced by genetic, demographic, and environmental factors [5,18,22,23]. Josephson et al. found mostly low titers in US O group SDP donors [1]. Our younger donors maintain strong antibodies [11,18]. Vegetarian diets may increase anti-A IgM titers due to plant antigens [5], while titration method variations could explain these differences [1,23,24].

Female donors showed higher antibody levels than males for anti-A IgM/IgG and anti-B IgG, consistent with studies [11,18]. This may be due to estrogen-enhanced B-cell activity and pregnancy-related antigen exposure [12,14]. Saidin et al. in their study (total n=311) found that female O donors (n=126) had higher antibody titers than males, with gender correlating with anti-B IgM titer (p = 0.001) and IgG titer (p = 0.002) [11]. Shah et al. observed low IgG titers in males and high IgM titers in females [25], though results warrant caution due to gender imbalance. Further balanced studies are needed.

Anti-A antibodies (IgM/IgG) and anti-B (IgG) showed no age-related changes, while anti-B IgM decreases with age, as shown by median titers (32 → 16). Tendulkar et al. found donors aged 18 to 29 had higher anti-B titers (p < 0.01), with females showing higher anti-A and anti-B (p < 0.001) [18]. Saidin et al. found decreasing median titers in older groups, significant in our study (p < 0.05) but not theirs (p > 0.05). Antibody titers were high in young females <40 years, with gender correlating with anti-B IgM (p = 0.001) and IgG titers (p = 0.002) [11]. These results reflect age-related changes in immune system function, particularly immunosenescence, reducing B-cell function and naturally occurring IgM antibodies [8]. The IgG antibodies remain stable due to maintenance by memory B cells and are less affected by aging [7,8]. Similar findings show IgM titers decrease with age while IgG levels remain constant [4,9].

This study compared antibody titers between 51 vegetarian and 49 mixed-diet donors. Around 56.9% of vegetarians had anti-A IgM ≥64 versus 28.6% of mixed-diet donors (p = 0.004), while anti-B IgM showed a non-significant trend. No IgG differences were observed. Results suggest vegetarian diets may elevate antibody titers. Kannan et al. reported higher titers in vegetarians, with 61.3% showing high titers (≥1:64) versus 48% of mixed-diet individuals (n = 454). Our findings indicate that diet influences antibody titers. Vegetarian diets rich in plant carbohydrates can modify gut microbiota composition, potentially influence antigenic exposure, and lead to higher IgM responses [6,7,9,10].

Smoking was significantly associated with reduced IgM and IgG levels compared to non-smokers (p < 0.05). Cigarette smoke impairs immune system function by reducing B-cell function, antibody production, and class switching. Research shows smoke exposure decreases bone marrow B220+CD43− B cells and affects the CD4+:CD8+ T-cell ratio, explaining the altered antibody production in smokers [26]. Chronic smoke exposure disrupts cytokine networks regulating humoral immunity [27]. Alcohol consumption was associated with lower anti-A/B IgG (p < 0.02) but did not significantly affect IgM (p > 0.3). Smoking reduced IgG levels (p = 0.02), and according to Tarbiah et al., smokers had lower IgG and higher IgM than non-smokers (p < 0.0001) [27]. In contrast, Arinola et al. reported elevated IgG and IgM in smokers and alcoholics, both statistically significant (p < 0.05), suggesting lifestyle effects may vary across populations [28].

Mikame et al. found negative correlations between BMI and antibody titer (p < 0.01) in type O blood donors. Donors with higher BMI had low antibody titers [8]. In our study, the correlation between BMI and antibody titers was not statistically significant. Elevated BMI may diminish antibody titers due to chronic inflammation and altered B-cell function, which can impair antibody production and class switching. Adipose tissue, particularly when in excess, secretes pro-inflammatory cytokines and adipokines, thereby creating a state of low-grade systemic inflammation. This persistent inflammatory environment can disrupt normal immune responses, including the production and maintenance of antibodies [8,9].

Spearman analysis showed no significant association between platelet yield and ABO antibody titers (anti-A IgM, anti-A IgG, anti-B IgM, anti-B IgG), with correlation coefficients near zero and p > 0.05. This indicates high-yield platelet units do not carry higher antibody titers and can be considered safe for transfusion below critical thresholds. Results show ABO antibody titers do not influence platelet yield in apheresis donors [3,5,14,17]. While high-yield platelet units contain larger plasma volumes with ABO antibodies, no correlation exists between yield and antibody titers [1,2,14]. Mitigation strategies like donor screening and platelet additive solutions reduce this risk, with antibody screening being more economical [3,14]. These results indicate that gender, diet, and lifestyle impact ABO antibody levels, while age and platelet yield have minimal influence.

Limitations

The sample size was limited to 100 donors from a tertiary care blood center, with predominantly male donors (90%) and fewer females (10%), which may have influenced antibody titers. Antibody titers were assessed using the conventional tube agglutination method, which is subject to observer variability and has lower reproducibility compared to gel card or automated techniques. Future studies could consider adopting gel cards or automated methods to enhance standardization and reproducibility. Since lifestyle factors were self-reported, recall bias and social desirability bias could affect data accuracy, particularly for alcohol and smoking habits. Clinical outcomes in recipients were not evaluated, preventing a correlation between donor antibody levels and transfusion reactions. Future research should aim to correlate donor antibody titers with recipient hemolysis outcomes to better define clinically significant thresholds.

## Conclusions

This study examines anti-A and anti-B antibody titers in O blood group SDP and their association with donor characteristics. A significant proportion of group O SDP donors showed elevated antibody titers, with anti-A IgM and IgG elevated in 43% and 56% of donors, respectively, and anti-B IgM and IgG elevated in 26% and 38%, respectively, with anti-A titers higher than anti-B, comparable to previous studies. These findings highlight the importance of antibody titer measurement for non-identical ABO transfusions. Associations were observed with age, gender, diet, and smoking; younger donors, females, vegetarians, and non-smokers tended to have higher titers. However, the modest sample size (n = 100) and limited number of female participants (n = 10) warrant cautious interpretation.

Larger, more representative studies are needed to clarify the influence of demographic and lifestyle factors. Routine evaluation of antibody titers in O group SDP could enhance platelet availability for emergency transfusions when ABO-identical units are not available, potentially reducing waiting times. This study highlights the need for standardized testing protocols and defined cutoff values to ensure transfusion safety, and further research is required to validate these findings in larger populations and to assess the clinical impact of low-titer O group platelet transfusion.

## Figures and Tables

**Figure 4:**
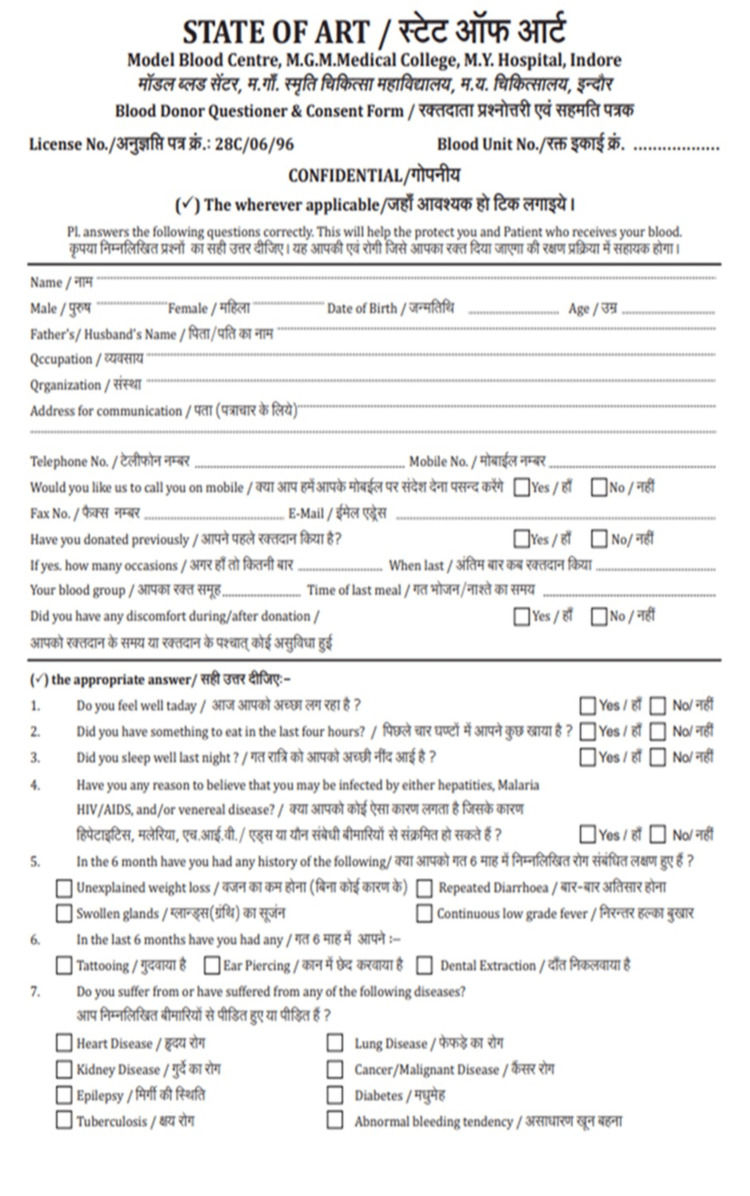
Page one of the blood donor consent and questionnaire form

**Figure 5:**
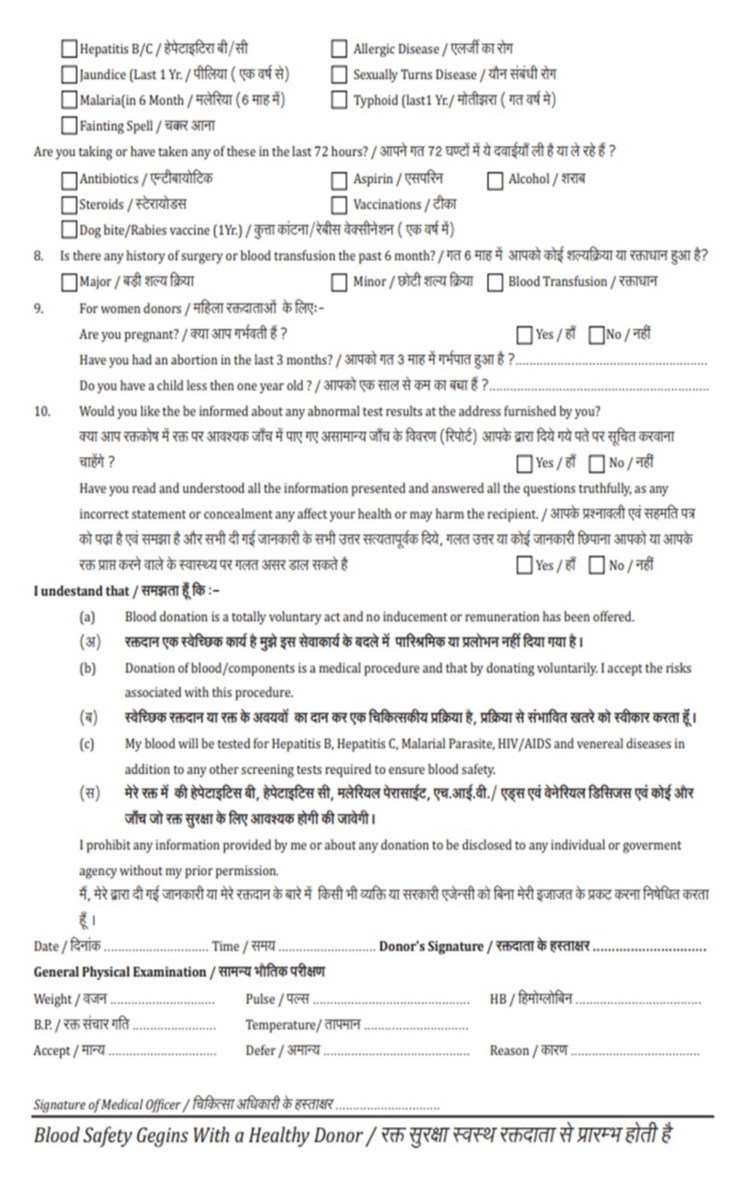
Page two of the blood donor consent and questionnaire form

## Appendices

Appendix A

Figures 4-5 feature the blood donor consent and questionnaire form provided to the study participants.

Appendix B

Listed below is the questionnaire used to collect data on demographic details, physical and lifestyle factors, and dietary habits for this study. 

Blood Donor Questionnaire: Physical and Lifestyle Factors

Section A: Demographic Information

Full Name:

Age (in years): ______

Sex: ☐ Male ☐ Female ☐ Other

Height (cm): ______

Weight (kg): ______

Body Mass Index (BMI): ______

Blood Group: ______

Section B: Physical Health

Do you have any chronic medical conditions (e.g., diabetes, hypertension, thyroid disorder)? ☐ Yes ☐ No If yes, please specify: ____________________

Are you currently taking any regular medications? ☐ Yes ☐ No If yes, please specify: ____________________

How often do you exercise or engage in physical activity? ☐ Daily ☐ 3-4 times/week ☐ 1-2 times/week ☐ Rarely/Never

How would you describe your overall fitness level? ☐ Excellent ☐ Good ☐ Average ☐ Poor

Section C: Lifestyle Habits

1.     Do you smoke tobacco? ☐ Yes, regularly ☐ Occasionally ☐ No, never If yes, number of cigarettes per day: ______ Duration (in years): ______

2.     Do you consume alcohol? ☐ Yes, regularly ☐ Occasionally ☐ No, never If yes, average drinks per week: ______ Duration (in years): ______

3.     Do you consume caffeinated drinks (tea, coffee, energy drinks)? ☐ Yes, regularly ☐ Occasionally ☐ No

4.     Do you use any other substances (e.g., recreational drugs)? ☐ Yes ☐ No

Section D: Dietary Habits

How often do you consume meat/non-vegetarian food? ☐ Daily ☐ Weekly ☐ Occasionally ☐ Never (Vegetarian)

Do you include dairy products in your diet? ☐ Yes, regularly ☐ Occasionally ☐ Never

Do you consume fruits and vegetables daily? ☐ Yes ☐ No

Do you take any nutritional supplements (e.g., multivitamins, protein powders)? ☐ Yes ☐ No If yes, please specify: ____________________

Name:

Signature:

Date:
